# Evolutionary history of the endemic water shrew *Neomys anomalus*: Recurrent phylogeographic patterns in semi‐aquatic mammals of the Iberian Peninsula

**DOI:** 10.1002/ece3.4487

**Published:** 2018-09-27

**Authors:** Marina Querejeta, Jose Castresana

**Affiliations:** ^1^ Institute of Evolutionary Biology (CSIC‐Universitat Pompeu Fabra) Barcelona Spain; ^2^ Bavarian State Collection of Zoology München Germany

**Keywords:** contact zones, mitochondrial genetic diversity, phylogeography, species distribution modeling

## Abstract

The Cabrera's water shrew (*Neomys anomalus*) is a small semi‐aquatic mammal whose taxonomic status was recently elevated from subspecies to species; as a consequence of this change, this species is now endemic to the Iberian Peninsula. In this study, we looked at its evolutionary history by combining phylogeography, the spatial distribution of genetic diversity, and species distribution modeling. To perform these analyses, we used noninvasive samples collected across the species distribution range and sequenced partial mitochondrial cytochrome *b* and D‐loop genes. Maximum‐likelihood and Bayesian phylogenetic trees derived from these sequences indicated that *N. anomalus* is divided into two main phylogroups that correlate strongly with geography, with two contact zones between the groups that showed limited spatial mixing between them. River basins were responsible for only a small percentage of the structure of the genetic diversity of this species despite its riparian habitat. The nucleotide diversity variation map showed the highest genetic diversity to be in the north of the Iberian Peninsula. Finally, species distribution modeling allowed the inference of an optimal area during the Last Interglacial in the north of the Iberian Peninsula, and multiple glacial refugia during the Last Glacial Maximum. The phylogeographic pattern of *N. anomalus* is strikingly similar to that of another semi‐aquatic Iberian mammal, the Pyrenean desman (*Galemys pyrenaicus*), revealing how Pleistocene glaciations could have had equivalent effects on species of similar ecology and distribution. This phylogeographic structure is consistent with *N. anomalus* having been isolated for long periods in multiple glacial refugia within the Iberian Peninsula, in agreement with the “refugia‐within‐refugia” hypothesis, and further supporting its status as a distinct species.

## 1 INTRODUCTION

The Iberian Peninsula was one of the most important glacial refugia in Europe during the Pleistocene (Hewitt, 1999, 2000). This southern European peninsula is geographically very complex, as it includes several large mountain ranges and covers a wide diversity of climates. It therefore has a large variety of habitats in a relatively small region, making it likely that there were several Iberian refugia for different plant and animal species (Gomez & Lunt, 2007). This scenario of multiple glacial refugia within the Iberian Peninsula (and other southern European peninsulas), known as the “refugia‐within‐refugia” hypothesis (Gomez & Lunt, 2007), has been confirmed for several Iberian endemics (Barbosa et al., 2017; Gonçalves et al., 2009; Igea et al., 2013; Martinez‐Solano, Teixeira, Buckley, & García‐París, 2006). The presence of genetic structures generated by past glaciations in various species from such a small region has enabled promising comparative phylogeographic studies to be undertaken aiming to reveal common refugia, postglacial dispersal patterns, and secondary contact zones (Avise, 2009; Moritz et al., 2009; Taberlet, Fumagalli, Wust‐Saucy, & Cosson, 1998).

The Cabrera's water shrew (*Neomys anomalus* Cabrera, 1907) is a small semi‐aquatic mammal belonging to the family Soricidae. It inhabits riparian ecosystems although it is also able to reach locations far from riverbanks (Rychlik, 1997). The species has several conspicuous morphological adaptations to the aquatic environment, such as large hind feet and the presence of short hairs in the tail and along the sides of the toes, which improve its swimming capacity, as well as a short snout to look for food in the river environment (Kryštufek, Davison, & Griffiths, 2000; Rychlik, 1997; Tapisso, Ramalhinho, Mathias, & Rychlik, 2013). This species was described from Spain by Cabrera (1907) but, from 1944 onwards, it was treated as a subspecies of the European form (Bobrinskiy, Kuznetsov, & Kuzyakin, 1944; Wilson & Reeder, 2005). However, initial mitochondrial data showed that the Iberian lineage was deeply divergent from the rest of specimens analyzed (Castiglia, Annesi, Aloise, & Amori, 2007). A more recent species delimitation study of the genus based on multiple nuclear markers revealed a clear genetic differentiation of *N. anomalus*, leading to it being considered a distinct species, separate from its sister species of European distribution *N. milleri* (Igea, Aymerich, Bannikova, Gosálbez, & Castresana, 2015); this elevated both taxa to species status, as originally described (Cabrera, 1907; Mottaz, 1907). *N. anomalus* is therefore endemic to the Iberian Peninsula, inhabiting the northern half as well as some isolated patches in the south (Ventura, 2007). Although it is considered to be of Least Concern according to the International Union for Conservation of Nature (IUCN), the current populations are suspected to be decreasing and its patchy distribution makes it susceptible to local extinction (Hutterer et al., 2008).

Despite the interest of this endemic water shrew, no previous studies have been carried out on the evolutionary history of *N. anomalus*. Being a semi‐aquatic species, it is more challenging to study, as factors such as the configuration of the river networks may play an important role in shaping its genetic structure (Byrne, Quintana, Bolkovic, Cassini, & Túnez, 2015; Igea et al., 2013; Querejeta, Fernández‐González, Romero, & Castresana, 2017; Vignieri, 2005). Additionally, Pleistocene glaciations could have affected its distribution and genetic structure by generating isolated populations in glacial refugia within the Iberian Peninsula (Gomez & Lunt, 2007). In this sense, it would be particularly interesting to compare the genetic structure of *N. anomalus* with that of the Pyrenean desman (*Galemys pyrenaicus*), another semi‐aquatic mammal endemic to the Iberian Peninsula (Palmeirim & Hoffmann, 1983), which was found to have a strong phylogeographic structure (Escoda, González‐Esteban, Gómez, & Castresana, 2017; Igea et al., 2013; Querejeta et al., 2016, 2017). As both species have a similar distribution and habitat, comparing their phylogeographic patterns could provide new insights into the biogeographical forces behind the origin and distribution of the Iberian fauna.

In this work, we aim to study the evolutionary history of the Cabrera's water shrew by addressing the following questions: (a) Is there some kind of genetic structure in this Iberian endemic species? (b) If so, did river basins play a role in shaping this genetic structure? (c) Which were the potential refugia during past glacial cycles? and (d) Is there a common phylogeographic pattern with the similarly distributed Pyrenean desman? To achieve these aims, we combined a phylogeographic and genetic diversity approach with species distribution modeling, as the combination of these techniques may provide a better picture of evolutionary scenarios (Graham, Ron, Santos, Schneider, & Moritz, 2004).

## 2 METHODS

### 2.1 Samples

We used 132 samples of *N. anomalus* collected from across its distribution range (Supporting Information Table S1). A total of 124 samples were obtained from excrements that water shrews deposit on emerged rocks of rivers, most of them collected between 2009 and 2015 while carrying survey works for *G. pyrenaicus* (Igea et al., 2013; Querejeta et al., 2017). In order to avoid using more than one fecal sample from the same individual, we only used samples with identical haplotypes if they were collected at least one kilometer apart, which is more than the typical home range of *Neomys* species (Cantoni, 1993; Lardet, 1988). We also used tissue samples of two specimens found dead in the field. Finally, the sampling was complemented with six DNA samples from a previous study (Igea et al., 2015).

### 2.2 DNA extraction and amplification

DNA was extracted from all samples using the DNeasy Blood and Tissue Kit from QIAGEN, following the manufacturer's instructions except that the elution volume was 50 μl. All extractions were carried out in a separated UV‐irradiated area with dedicated equipment. We then amplified 752 bp of the mitochondrial cytochrome *b* gene using primers from a previous study (Igea et al., 2015). In addition, we designed, with the help of primer3plus (Untergasser et al., 2012), forward and reversed primers to sequence a mitochondrial D‐loop fragment of approximately 450 bp: ATCAACACCCAAAGCTGATATTCTA, in the tRNA‐Pro gene, and TTAATGTGCCTTGTCCGATT, in the D‐loop. This gene was amplified in 103 of the samples. All PCRs, including necessary controls, were set up in a dedicated PCR clean room that is physically separated from post‐PCR working areas and regularly decontaminated by UV irradiation. PCR conditions were as in Igea et al. (2015). PCR products were checked in agarose gels, purified with ExoSAP‐IT, and sequenced in Macrogen Inc (Seoul, South Korea). The sequences obtained were edited and assembled with geneious pro (Biomatters Ltd).

### 2.3 Phylogenetic analysis

mafft v7.130b (Katoh & Standley, 2013) was used to align the D‐loop fragment with an iterative refinement method (options: –maxiterate 1000 –localpair). Due to the complexity of the D‐loop in shrews (Fumagalli, Taberlet, Favre, & Hausser, 1996; Sbisà, Tanzariello, Reyes, Pesole, & Saccone, 1997), there were many gaps in both end regions. Therefore, gblocks (Castresana, 2000) was used to eliminate gap positions, which left a common central region of 272 bp. Finally, the cytochrome *b* and D‐loop alignments were concatenated into a 1,024 bp alignment, leaving missing data for those specimens in which only the cytochrome *b* was obtained.

A maximum‐likelihood tree was generated from the concatenated genes using raxML version 8 (Stamatakis, 2006). We first chose the best partition and substitution model scheme for the concatenated genes using the software partitionfinder version 1.1.1 (Lanfear, Calcott, Ho, & Guindon, 2012). We defined four possible partitions: one for each codon position of the cytochrome *b* and a fourth one for the D‐loop. We selected the option for raxML, the Bayesian information criterion, branch lengths linked, and all partition schemes analyzed. The following substitutions models were selected: GTR+G for the first and second codon positions, and GTR+I+G for the third codon position and the D‐loop. Since raxML uses a single model for all partitions, we selected GTR+I+G, but similar results were obtained with GTR+G (not shown). The best tree was estimated using 100 random starting trees. Bootstrap support values were calculated in a separate run using the rapid bootstrap option with 100 replications. The tree was rooted at the midpoint for the figure.

A Bayesian phylogenetic tree was conducted using beast v1.8.4. (Drummond & Rambaut, 2007). To select a statistically appropriate model, a Bayes factor approach was used (Baele et al., 2012). The marginal likelihood of each model was calculated using path sampling and stepping‐stone sampling with 100 path steps of 10,000,000 generations. Markov chain Monte Carlo analyses consisted of 10,000,000 generations sampled every 1,000 generations. Convergence was checked with tracer v.1.6 (part of the beast package). Different options were tested within each parameter category, and a more complex model was selected only if it converged in different runs and the improvement in the log marginal likelihood was greater than 5 (Baele et al., 2012). Using these criteria, the selected model was a HKY substitution model with empirical base frequencies and Gamma site heterogeneity for each partition (unlinked substitution models), a strict clock model for all partitions (linked clock model), and a coalescent constant size tree model. The final analysis with this model setup was based on 100,000,000 generations. The summary tree was generated after removing 10% generations as burn‐in with treeannotator (beast package), using median heights.

### 2.4 Genetic diversity

Variation of nucleotide diversity across space was calculated from the cytochrome *b* , which was sequenced in all samples. Using the alignment of the 132 cytochrome *b* sequences and the sample coordinates, we computed, for each sampling point, the nucleotide diversity values (*π*) of samples found in a radius of 75 km around it (Igea et al., 2013). For each sample point, only samples of the same phylogroup were used for the calculation, and only values based on five samples or more were considered. Color‐coded nucleotide diversity values were then plotted on a map. Similar maps were found for other radius (between 25 and 75 km) or using the Ø estimator of nucleotide diversity (not shown). Diversity values were obtained with the Bioperl library PopGen (Stajich & Hahn, 2005).

We performed a one‐way analysis of molecular variance (AMOVA) to calculate the amount of nucleotide diversity that can be explained by river basins (Supporting Information Table S1). We first computed a matrix of pairwise genetic distances from the cytochrome *b* sequences using the R package ape (Paradis, 2004). The AMOVA was then performed with the R package pegas (Paradis, 2010), treating river basin as unique factor.

### 2.5 Species distribution modeling

We compiled occurrence data for *N. anomalus* within its recently delimited distribution (Igea et al., 2015) using the Global Biodiversity Information Facility (Flemons, Guralnick, Krieger, Ranipeta, & Neufeld, 2007) and the sample localities of this study. To avoid unbalanced sampling, we selected one sample for each 1 × 1 km grid cell, leaving a final dataset of 858 localities.

The environmental data were obtained from WorldClim 1.4 (Hijmans, Cameron, Parra, Jones, & Jarvis, 2005), using a resolution of 2.5 arc‐minutes, for both current and past conditions that included the Last Glacial Maximum (~22,000 years ago) and the Last Interglacial (~120,000–140,000 years ago). Three different general atmospheric circulation models of the Last Glacial Maximum were used: CCSM, MIROC, and MSPI. All layers were cropped to the extent of the Iberian Peninsula using the R package *raster* (Hijmans et al., 2016). After computing the Pearson's correlation matrix between all bioclimatic variables, 11 variables with a correlation lower than 0.8 were kept: Annual Mean Temperature (BIO1), Mean Diurnal Range (BIO2), Isothermality (BIO3), Minimum Temperature of Coldest Month (BIO6), Temperature Annual Range (BIO7), Mean Temperature of Wettest Quarter (BIO8), Mean Temperature of Driest Quarter (BIO9), Mean Temperature of Warmest Quarter (BIO10), Precipitation of Wettest Month (BIO13), Precipitation of Driest Month (BIO14), and Precipitation Seasonality (BIO15).

We generated the species distribution model using maxent 3.3.3k (Phillips, Anderson, & Schapire, 2006). The model was replicated 50 times, selecting training and test samples randomly. All other settings were left as default. The obtained area under the curve (AUC) value of 0.841 indicated a good performance of the model (Phillips & Dudik, 2008). The average model estimated was then projected onto the Last Interglacial and the three Last Glacial Maximum models. To show a consensus of the three Last Glacial Maximum projections, the output of each model was set to logistic, a binary layer using different percentile thresholds was created for each model, and the consensus was obtained with the Raster Calculator tool of qgis 2.0.1 (QGIS Development Team, 2013). The use of a standard 10th percentile generated a consensus covering most of the studied area (not shown), while a more restricted 90th percentile was more informative at the scale of the Iberian Peninsula.

## 3 RESULTS

### 3.1 Phylogenetic analyses

**Figure 1:**
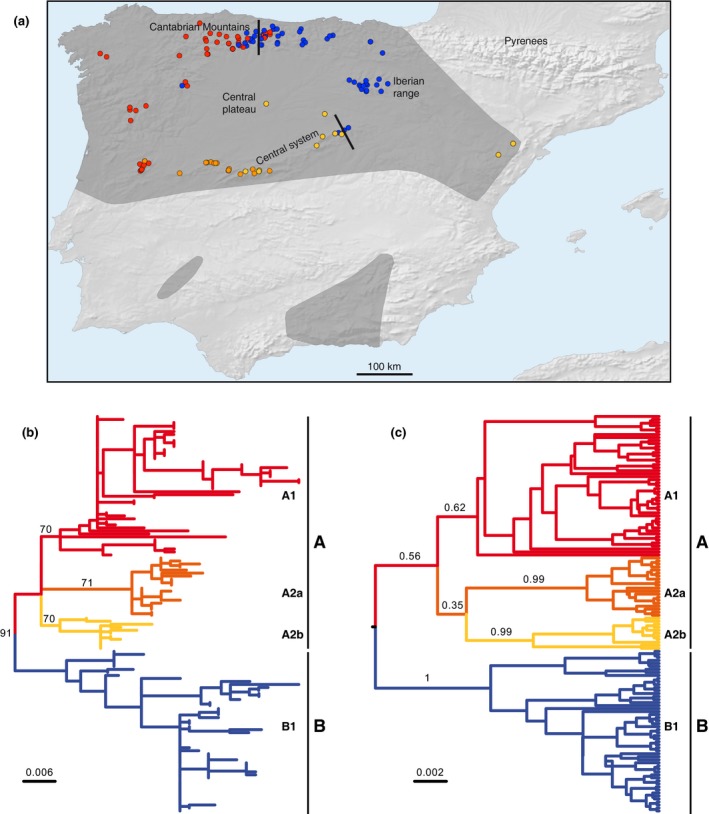
Phylogeography of *Neomys anomalus* with the main mitochondrial groups shown in different colors. (a) Map showing the distribution of the mitochondrial phylogroups with two lines indicating the locations of the contact zones between the main groups, A and B. The dark gray area represents the species distribution range. Names of the main geographical terms mentioned in the text are shown. (b) Maximum‐likelihood phylogenetic tree with midpoint rooting of the cytochrome *b* and D‐loop concatenated sequences showing the percent bootstrap support of the main nodes and the scale in substitutions per position. (b) Bayesian tree of the same sequences with the posterior probability values of the main nodes

The map in Figure 1a shows the distribution of the samples, which preferentially cover mountainous areas, where the abundance of small rivers facilitates sampling excrements of the species. Both the maximum‐likelihood (Figure 1b) and Bayesian (Figure 1c) trees showed two main phylogroups, A and B, strongly correlated with geography: A is distributed in the western half and B in the northeastern part of the range (Figure 1a and Supporting Information Figure S1). The bootstrap value for this split in the maximum‐likelihood tree was 91%. In the Bayesian tree, the root coincided with the split of both groups and the posterior probability was 0.56 for group A and 1 for group B. The divergence between both lineages (p distance) was 1.16% for the cytochrome *b* fragment. Two contact zones between the two phylogroups were detected, one in the Cantabrian Mountains and the other in the northeast of the Central System (Figure 1a). Additionally, group A can be subdivided into three subgroups (A1, A2a, and A2b) forming an almost unresolved trichotomy but each of them with moderate support both in the maximum‐likelihood (70%, 71%, and 70% bootstrap values, respectively) and Bayesian (0.62, 0.99, and 0.99 posterior probability values, respectively) trees (Figure 1). They also have a clear geographical segregation: group A1 occupies the northwestern area, whereas groups A2a and A2b occur in the occidental and oriental parts of the Central System, respectively. The assignment of each sample to either group A1, A2a, A2b, or B was identical for both trees (Supporting Information Table S1), supporting the genetic structure found. As expected for coalescent trees, all other internal groupings within the tree have very low support values.

### 3.2 Genetic diversity

**Figure 2:**
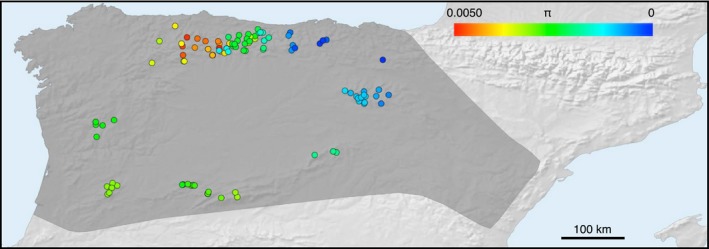
Map showing mitochondrial nucleotide diversity around each sampling point. The upper scale indicates nucleotide diversity (*π*) values

Genetic diversity was higher for the A phylogroup than for the B using different estimators (Supporting Information Table S2). To recover more resolution in the variability of genetic diversity across space, we calculated mitochondrial nucleotide diversity in each sampling point. The color‐coded plot showed strong differences within the distribution range (Figure 2). The highest value was 0.0050 and the lowest 0.0003. The area of highest genetic diversity values was located in the north and, particularly, in the north‐central part of the Iberian Peninsula. Since genetic diversity was calculated in each sampling point for samples of the same phylogroup (A1, A2a, A2b, or B), nucleotide diversity is not inflated in the contact zones due to mixing of phylogroups. The genetic diversity was lower in the eastern populations.

The one‐way AMOVA using the ten river basins in which the samples were collected as factor showed that only 19% of the variation in genetic diversity was due to isolation in river basins.

### 3.3 Species distribution modeling

**Figure 3:**
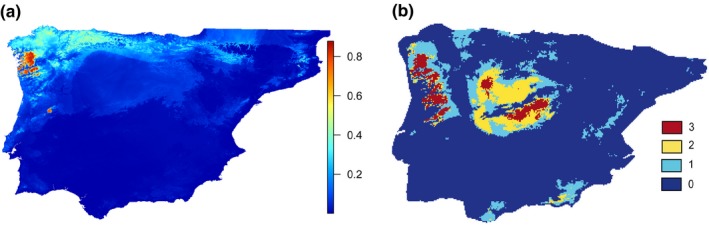
(a) Projection of the species distribution model into the Last Interglacial with the scale indicating predicted probability of presence. (b) Areas predicted by the three Last Glacial Maximum models, using the 90th percentile values. Colors indicate areas predicted by three models (red), two (yellow), or only one model (light blue)

The potential distribution for the present time matched the actual species range, except that the southern patches and the eastern area showed a low probability (Supporting Information Figure S2A). The potential distribution for the Last Interglacial (Figure 3a) showed the north of the Iberian Peninsula to be a favorable area, with the highest probability in a small area in the northwest. In contrast, the optimal surface increased substantially in the Last Glacial Maximum for the three climatic scenarios (Supporting Information Figure S2). The consensus of the three models (Figure 3b) showed two large potential optimal regions, one in the western region of the main *N. anomalus* distribution range and another in the Central System, and a third, smaller area in the Central Plateau.

## 4 DISCUSSION

### 4.1 Phylogeography of the Cabrera's water shrew (*Neomys anomalus*)

This study is the first to attempt to explain the evolutionary history of *N. anomalus* using mitochondrial data after it was recently recognized as species and, consequently, an Iberian endemism (Igea et al., 2015). With regard to the phylogeographic pattern observed, the four main phylogroups detected, A1, A2a, A2b, and B, are mainly allopatric (Figure 1). Designation of subgroups A2a and A2b as part of a common group, A2, is proposed here to match a similarly distributed mitochondrial group in *G. pyrenaicus* (Igea et al., 2013), but further data will be necessary to corroborate it because in the present trees they are part of an unresolved trichotomy. Group A1 is mostly distributed in the western half, group A2 in the Central System, and group B occupies the northeastern part of the distribution (with only one sample of group B in the area of group A, in the northwest of the Central Plateau). The two main contact zones between the mitochondrial lineages A and B, situated in the Cantabrian Mountains and the Central System, respectively, are well delimited, although they show different levels of spatial mixing. In the Cantabrian Mountains contact zone, lineages A and B coexist in many rivers. The Central System contact zone is stricter, as samples of both groups were detected simultaneously in only one river, although the scant sampling in this area does not allow us to determine whether samples of the two groups could be present in other rivers. Isolated patches in the south as well as peripheral populations in the east, where the species is scarce (Torre & Tella, 1994; Ventura, 2007), are interesting populations but, unfortunately, sampling was limited in them. Further studies should be carried out on these populations to understand whether they are due to recent expansions of the phylogroups detected here or to isolation in ancient refugia. There is also very little sampling in the Central Plateau, where mixing of phylogroups or other contact zones could be detected with additional sampling. In any case, the strong mitochondrial structure revealed here for *N. anomalus* indicates that the Iberian populations of this water shrew are not the product of a recent expansion from Europe; rather, these results support that the *N. anomalus* lineage evolved independently in the Iberian Peninsula (Igea et al., 2015).

The highest levels of mitochondrial genetic diversity in *N. anomalus* were found in the northern part of its distribution (Figure 2). Interestingly, this northern region was a favorable habitat for *N. anomalus* during the Last Interglacial (~120,000–140,000 years ago; Figure 3), which is consistent with the continuous existence of the species for a long time period in the north. In contrast, the southern and eastern parts of the main distribution area have lower levels of genetic diversity (Figure 2), suggesting that these areas could have been colonized more recently. Even so, the match between the genetic diversity map and the Last Interglacial prediction is far from perfect as the maximum genetic diversity is located in the center of the Cantabrian Mountains, whereas the most favorable area during the Last Interglacial was in the northwest.

The species distribution models for the Last Glacial Maximum are consistent in showing the western area of the Iberian Peninsula as an optimal region for *N. anomalus*. This is largely coincident with the major refugium predicted for *G. pyrenaicus* (Igea et al., 2013; Querejeta et al., 2017). There is another optimal area situated in the Central System and a minor one in the Central Plateau. These models are compatible with multiple refugia predicted for the Last Glacial Maximum and the differentiation of the major phylogroups of *N. anomalus* in them, as predicted by the “refugia‐within‐refugia” hypothesis (Gomez & Lunt, 2007). However, the optimal areas inferred for the Last Glacial Maximum are difficult to reconcile with the map of genetic diversity variation (Figure 2). This indicates that glacial refugia do not always lead to areas of current high genetic diversity. This could be due to complex scenarios involving local extinctions, recolonizations, and expansions from refugia to other areas during the Holocene (Godinho, Crespo, & Ferrand, 2008; Querejeta et al., 2017). In addition, the possibility of mitonuclear discordance makes that nuclear data may be necessary to reveal further details on the glacial history of the species (see below).

Although *N. anomalus* is a semi‐aquatic species associated with riparian ecosystems, river basins contribute relatively little to its genetic structure (19%). This value is even lower than that found for *G. pyrenaicus* (Igea et al., 2013). This result suggests that *N. anomalus* is able to disperse overland to even a greater extent than *G. pyrenaicus* (Querejeta et al., 2017), as expected due to the lower dependency of water shrews on the aquatic ecosystem (Rychlik, 1997).

### 4.2 Concordant phylogeographic patterns in two semi‐aquatic mammals of the Iberian Peninsula

The concordance of genealogical patterns in codistributed species of similar ecologies is fundamental to understanding how biogeographical forces molded flora and fauna in different regions of the world (Avise, 2009; Moritz et al., 2009; Taberlet et al., 1998). Interestingly, both *N. anomalus* and *G. pyrenaicus* are mainly distributed in the north of the Iberian Peninsula and are frequently found in the same rivers, usually in mountainous areas, where the habitat of both species is more abundant and better preserved (Nores, Queiroz, & Gisbert, 2007; Ventura, 2007). Similar to *N. anomalus*, the mitochondrial phylogenetic tree of *G. pyrenaicus* showed two major phylogroups (also designated A and B) of equivalent divergence and two contact zones between them (figure 1 in Igea et al., 2013). There is also a striking correspondence between the distributions of the main mitochondrial phylogroups and their contact zones in these two species. One of the contact zones described for *G. pyrenaicus* is situated in the Cantabrian Mountains, in basically the same position as that of *N. anomalus*. The other contact zone is located in the Iberian Range, to the north of the equivalent contact zone shown here for *N. anomalus*, but not far from it. Additionally, each *G. pyrenaicus* phylogroup was subdivided into two subgroups, giving rise to four groups of allopatric distribution: A1, A2, B1, and B2. The distributions of the A1, A2, and B1 groups are very similar in both species. Nonetheless, there are also important differences between the phylogeographic patterns of the two species. In the case of *G. pyrenaicus*, both contact zones between the A and B mitochondrial lineages were stricter, with basically no spatial mixing between the phylogroups, in contrast with a relatively greater degree of mixing in *N. anomalus*. Furthermore, from the common distribution nucleus of both species, *G. pyrenaicus* expanded to the Pyrenees into a phylogroup not coincident with *N. anomalus,* whereas *N. anomalus* did it toward the south and east. Despite these differences, the notable concordance of the distributions of three of the major phylogroups and the locations of their contact zones suggest that glacial cycles had a concomitant impact on these two semi‐aquatic species. The equivalence of the principal contact zones is likely due to secondary contacts after postglacial recolonizations from the same or nearby refugia. The results obtained using mitochondrial data from both species highlight the fundamental effect that glacial cycles had on Iberian species, including semi‐aquatic ones, and help us better understand the origin and distribution patterns of Iberian endemics (Gomez & Lunt, 2007).

Genomic data used to analyze the population history of *G. pyrenaicus* were basically in agreement with its mitochondrial phylogeography but provided additional significant details, such as the population dynamics taking place in the contact zones (Escoda et al., 2017; Querejeta et al., 2016). It will be of interest to know how genomic data of *N. anomalus* complete the knowledge on the evolutionary history of this species since mitonuclear discordances are present in many species (Toews & Brelsford, 2012) and, therefore, mitochondrial data alone may not reveal the whole picture. Multinuclear data may also help to determine an adequate time frame for the origin and evolution of the main populations (Igea et al., 2015; Sánchez‐Gracia & Castresana, 2012). Obtaining a robust evolutionary scenario based on genomic data for *N. anomalus* and its comparison to that of *G. pyrenaicus* will help to shed further light on the geographical forces that shaped the genetic structure and the evolutionary history of these Iberian species.

## 5 CONCLUSIONS

After the split of the *N. anomalus* and *N. milleri* lineages around 0.40 Myr ago (Igea et al., 2015), we can envisage a scenario in which *N. anomalus* became isolated from its sister lineage in the north and northwest of the Iberian Peninsula, as suggested by the optimal areas predicted for the Last Interglacial projection (Figure 3a). Subsequently, the existence of at least two large potential glacial refugia during the Last Glacial Maximum (Figure 3b) could have played a role in genetically differentiating the main phylogroups (Figure 1). The occidental glacial refugium would have been the most important for the species. The position of this refugium, as well as the optimal area of the Last Interglacial in the northwest of the Iberian Peninsula, far from the distribution range of *N. milleri*, supports the existence of a long period during which these two species evolved independently, and is in agreement with the low levels of gene flow between them (Igea et al., 2015). The phylogeographic pattern found here for *N. anomalus* is in remarkable concordance with that of *G. pyrenaicus* (Igea et al., 2013; Querejeta et al., 2016), a unique Iberian mammal with no other extant species in the genus (Wilson & Reeder, 2005), giving additional support to the fact that *N. anomalus* evolved separately from its sister species and thus reinforcing its species status (Igea et al., 2015).

Currently, the IUCN ranks the conservation status of the Cabrera's water shrew as Least Concern (Hutterer et al., 2008). However, this evaluation was made when *N. anomalus* and *N. milleri* were considered to belong to the same species (Wilson & Reeder, 2005). The species status of *N. anomalus*, supported by previous genetic analyses and the phylogeographic pattern revealed here, stresses the need to improve our knowledge of its populations. Although *N. anomalus* is abundant in favorable habitats, its restricted distribution, the vulnerability of its aquatic habitat, and the existence of peripheral and isolated populations with low densities of individuals may require a reassessment of its conservation status in specific regions.

## AUTHOR CONTRIBUTIONS

J.C. conceived the ideas and analyzed data; M.Q. performed the laboratory work and analyzed data; and M.Q. and J.C. led the writing.

## DATA ACCESSIBILITY

All sequences have been deposited in GenBank under accession numbers MH111152–MH111277 for the cytochrome *b* and MH111279–MH111381 for the D‐loop.
