# Supplementary material for: Evolutionary history of the endemic water shrew Neomys anomalus: Recurrent phylogeographic patterns in semi‐aquatic mammals of the Iberian Peninsula
Source: Ecol Evol. 2018 Sep 27;8(20):10138–46. doi: 10.1002/ece3.4487 (PMC6206195; doi:10.1002/ece3.4487)
Supplement: Supplementary file 1 [file ECE3-8-10138-s001.pdf]

SUPPORTING INFORMATION

Ecology and Evolution

Evolutionary history of the endemic water shrew *Neomys anomalus*: recurrent phylogeographic patterns in semi-aquatic mammals of the Iberian Peninsula

Marina Querejeta and Jose Castresana

Table S1. Samples used in this study with information about location, river basin, collection year, mitochondrial phylogroup, sample type and genes sequenced in each sample. Samples of a previous study (Igea et al. 2015) are also included.

Specimen Code	Latitude	Longitude	Basin	Year	Phylogroup	Sample type	Cytochrome *b*	D-loop
IBE-C326	43.10042	-5.0077	Duero	2009	B	Faeces	yes	yes
IBE-C331	43.19352	-4.90761	Deva	2009	A1	Faeces	yes	yes
IBE-C333	43.31652	-4.85735	Deva	2009	B	Faeces	yes	yes
IBE-C360	43.25681	-4.7649	Deva	2009	B	Faeces	yes	yes
IBE-C370	43.11025	-4.74269	Deva	2009	B	Faeces	yes	no
IBE-C385	43.22616	-5.03102	Sella	2009	B	Faeces	yes	no
IBE-C524	43.05728	-6.24462	Nalón	2009	A1	Faeces	yes	yes
IBE-C529	42.08666	-6.71346	Duero	2010	A1	Faeces	yes	yes
IBE-C544	43.10209	-5.80897	Nalón	2009	A1	Faeces	yes	yes
IBE-C568	42.98546	-6.00301	Duero	2009	A1	Faeces	yes	yes
IBE-C573	43.01	-6.22696	Miño	2009	A1	Faeces	yes	no
IBE-C597	42.97739	-4.18256	Ebro	2010	B	Faeces	yes	yes
IBE-C630	43.25778	-4.26971	Saja	2009	B	Faeces	yes	no
IBE-C637	43.20201	-4.29735	Saja	2009	B	Faeces	yes	no
IBE-C688	43.14253	-5.05624	Sella	2009	B	Faeces	yes	no
IBE-C690	43.12679	-5.03696	Sella	2009	A1	Faeces	yes	no
IBE-C692	43.25934	-4.80843	Deva	2009	B	Faeces	yes	yes
IBE-C698	43.14565	-4.91028	Deva	2009	A1	Faeces	yes	yes
IBE-C740	43.25385	-4.83628	Deva	2009	B	Faeces	yes	no
IBE-C748	43.18185	-4.99829	Sella	2009	A1	Faeces	yes	yes
IBE-C761	43.02841	-4.21989	Ebro	2009	B	Faeces	yes	no
IBE-C785	42.75127	-8.41541	Ulla	2010	A1	Faeces	yes	no
IBE-C825	41.58792	-7.80231	Duero	2010	A1	Faeces	yes	yes
IBE-C843	41.63747	-7.8033	Duero	2010	A1	Faeces	yes	yes
IBE-C1029	42.97746	-4.7547	Duero	2010	B	Faeces	yes	yes
IBE-C1054	42.10114	-2.90045	Ebro	2011	B	Faeces	yes	no
IBE-C1061	42.12813	-2.9273	Ebro	2011	B	Faeces	yes	yes
IBE-C1114	42.949	-4.75971	Duero	2010	B	Faeces	yes	no
IBE-C1144	43.10283	-6.64592	Nalón	2010	A1	Igea et al. 2015	yes	no
IBE-C1435	41.71436	-4.98809	Duero	2010	A2b	Igea et al. 2015	yes	no
IBE-C1510	40.33764	-5.76692	Tajo	2010	A2a	Faeces	yes	yes
IBE-C1515	40.3709	-5.75221	Tajo	2010	A2a	Faeces	yes	yes
IBE-C1516	42.08723	-6.72401	Duero	2010	B	Faeces	yes	yes
IBE-C1518	40.48633	-6.00621	Tajo	2010	A2a	Faeces	yes	yes
IBE-C1520	40.51167	-6.13109	Tajo	2010	A2a	Faeces	yes	yes
IBE-C1662	41.97124	-2.61497	Duero	2010	B	Igea et al. 2015	yes	yes
IBE-C1683	42.16723	-6.6566	Duero	2010	A1	Igea et al. 2015	yes	yes
IBE-C1750	40.34456	-5.76397	Tajo	2010	A2a	Faeces	yes	no
IBE-C1789	40.26076	-5.52136	Duero	2008	A2a	Igea et al. 2015	yes	no
IBE-C1856	43.20814	-5.19865	Sella	2010	B	Faeces	yes	no
IBE-C2520	43.10003	-5.57721	Nalón	2011	A1	Faeces	yes	yes
IBE-C2604	40.89207	0.1101	Ebro	2011	A2b	Faeces	yes	no

IBE-C2605	43.10958	-5.22665	Nalón	2011	B	Faeces	yes	no
IBE-C2664	40.66073	-0.19369	Ebro	2011	A2b	Igea et al. 2015	yes	yes
IBE-C2843	40.27592	-5.23156	Duero	2011	A2a	Faeces	yes	yes
IBE-C3230	41.67061	-7.46992	Duero	2011	A1	Faeces	yes	no
IBE-C3237	43.17402	-5.39107	Nalón	2011	B	Faeces	yes	yes
IBE-C3244	41.3685	-7.7885	Duero	2011	A1	Faeces	yes	yes
IBE-C3247	43.05733	-5.55589	Nalón	2011	A1	Faeces	yes	no
IBE-C3262	40.33895	-5.13038	Duero	2011	A2b	Faeces	yes	yes
IBE-C3271	41.57444	-7.67904	Duero	2011	A1	Faeces	yes	no
IBE-C3273	43.04699	-5.27944	Duero	2011	B	Faeces	yes	yes
IBE-C3330	40.3163	-5.41582	Duero	2011	A2b	Faeces	yes	yes
IBE-C3350	40.36109	-5.26672	Duero	2011	A2a	Faeces	yes	yes
IBE-C3394	43.16645	-4.65828	Deva	2011	B	Faeces	yes	yes
IBE-C3430	42.18963	-2.92848	Ebro	2011	B	Faeces	yes	no
IBE-C3456	42.22131	-2.65816	Ebro	2011	B	Faeces	yes	no
IBE-C3458	42.10397	-2.70577	Ebro	2011	B	Faeces	yes	no
IBE-C3463	42.12746	-2.47893	Ebro	2011	B	Faeces	yes	yes
IBE-C3493	43.17028	-6.14498	Nalón	2012	A1	Faeces	yes	yes
IBE-C3611	42.90949	-5.39537	Duero	2011	B	Faeces	yes	yes
IBE-C3617	42.97483	-5.55803	Duero	2011	A1	Faeces	yes	no
IBE-C3690	43.14796	-5.9559	Nalón	2012	A1	Faeces	yes	no
IBE-C3794	42.68339	-8.28367	Ulla	2013	A1	Faeces	yes	yes
IBE-C3946	43.07209	-5.02095	Duero	2012	A1	Faeces	yes	yes
IBE-C3947	43.05577	-5.09282	Duero	2012	B	Faeces	yes	yes
IBE-C4023	43.05696	-3.73039	Ebro	2012	B	Faeces	yes	yes
IBE-C4042	43.12651	-3.61119	Ebro	2012	B	Faeces	yes	no
IBE-C4090	41.50399	-3.77884	Duero	2013	A2b	Tissue	yes	yes
IBE-C4337	42.99514	-2.83592	Ebro	2014	B	Faeces	yes	yes
IBE-C4339	43.11491	-3.67615	Ebro	2014	B	Faeces	yes	yes
IBE-C4347	42.99513	-2.83591	Ebro	2014	B	Faeces	yes	yes
IBE-C4487	42.1603	-3.1695	Duero	2014	B	Faeces	yes	yes
IBE-C4499	42.09864	-3.06499	Duero	2014	B	Faeces	yes	yes
IBE-C4505	42.012	-3.01823	Duero	2014	B	Faeces	yes	yes
IBE-C4511	42.06634	-3.03681	Ebro	2014	B	Faeces	yes	yes
IBE-C4515	43.37716	-6.36027	Nalón	2015	A1	Tissue	yes	yes
IBE-C4618	41.03733	-3.8249	Duero	2015	A2b	Faeces	yes	yes
IBE-S1920	42.76235	-2.5866	Ebro	2009	B	Faeces	yes	no
VM77-31-B	42.20373	-3.27355	Duero	2014	B	Faeces	yes	yes
WM04-SO61-B	41.96772	-2.92292	Duero	2014	B	Faeces	yes	yes
WM04-SO63-B	41.9882	-2.9154	Duero	2014	B	Faeces	yes	yes
WM05-01-A	42.08181	-2.97969	Ebro	2014	B	Faeces	yes	yes
XX23-02-A	40.85048	-3.94726	Tajo	2014	A2b	Faeces	yes	yes
IBE-BC0112	42.70769	-6.78053	Miño	2014	A1	Faeces	yes	yes
IBE-BC0121	40.32854	-4.91813	Tajo	2014	A2a	Faeces	yes	yes
IBE-BC0201	40.50271	-6.2179	Duero	2014	A2a	Faeces	yes	yes
IBE-BC0209	42.84774	-5.67969	Duero	2014	A1	Faeces	yes	yes
IBE-BC0246	40.28092	-6.77457	Duero	2014	A2a	Faeces	yes	yes

IBE-BC0254	40.33267	-5.76922	Tajo	2014	A2a	Faeces	yes	yes
IBE-BC0304	43.0111	-5.30854	Duero	2014	B	Faeces	yes	yes
IBE-BC0357	40.50319	-6.21647	Duero	2014	A2a	Faeces	yes	yes
IBE-BC0359	42.8479	-5.68013	Duero	2014	A1	Faeces	yes	yes
IBE-BC0374	40.34232	-5.7647	Tajo	2014	A2a	Faeces	yes	yes
IBE-BC0396	42.9575	-5.76094	Duero	2014	A1	Faeces	yes	yes
IBE-BC0407	40.49979	-6.23006	Duero	2014	A2a	Faeces	yes	yes
IBE-BC0416	40.4869	-6.06411	Tajo	2014	A2a	Faeces	yes	yes
IBE-BC0530	40.49129	-6.03258	Tajo	2014	A2a	Faeces	yes	yes
IBE-BC0540	42.8479	-5.68013	Duero	2014	A1	Faeces	yes	yes
IBE-BC0589	40.31703	-5.13832	Duero	2014	A2b	Faeces	yes	yes
IBE-BC0592	42.84783	-5.68012	Duero	2014	A1	Faeces	yes	yes
IBE-BC0632	42.93044	-5.5607	Duero	2014	B	Faeces	yes	yes
IBE-BC0642	42.95808	-5.76149	Duero	2014	A1	Faeces	yes	yes
IBE-BC0662	42.96648	-5.42442	Duero	2014	A1	Faeces	yes	yes
IBE-BC0694	43.054	-5.32041	Duero	2014	B	Faeces	yes	yes
IBE-BC0708	42.84783	-5.68058	Duero	2014	A1	Faeces	yes	yes
IBE-BC0715	42.91698	-5.53824	Duero	2014	A1	Faeces	yes	yes
IBE-BC0716	42.96655	-5.42442	Duero	2014	B	Faeces	yes	yes
IBE-BC0720	43.06115	-5.37404	Duero	2014	A1	Faeces	yes	yes
IBE-BC0726	42.88462	-5.46132	Duero	2014	A1	Faeces	yes	yes
IBE-BC0750	42.99805	-5.25956	Duero	2014	B	Faeces	yes	yes
IBE-BC0781	42.84788	-5.68057	Duero	2014	A1	Faeces	yes	yes
IBE-BC0874	42.93563	-4.24247	Duero	2014	B	Faeces	yes	yes
IBE-BC0907	42.84849	-6.21617	Duero	2014	A1	Faeces	yes	yes
IBE-BC0908	42.73962	-6.20847	Miño	2014	A1	Faeces	yes	yes
IBE-BC0949	42.73718	-6.19317	Miño	2014	A1	Faeces	yes	yes
IBE-BC1027	42.92745	-5.08191	Duero	2014	B	Faeces	yes	yes
IBE-BC1072	42.92961	-5.11575	Duero	2014	B	Faeces	yes	yes
IBE-BC1330	40.48571	-7.41484	Mondego	2015	A1	Faeces	yes	yes
IBE-BC1339	40.38271	-7.54584	Tajo	2015	A2a	Faeces	yes	yes
IBE-BC1382	40.52997	-7.49779	Mondego	2015	A1	Faeces	yes	yes
IBE-BC1399	41.08402	-3.42982	Tajo	2015	A2b	Faeces	yes	yes
IBE-BC1405	40.46776	-7.59768	Mondego	2015	A1	Faeces	yes	yes
IBE-BC1414	41.11642	-3.49308	Tajo	2015	B	Faeces	yes	yes
IBE-BC1416	40.5303	-7.49747	Mondego	2015	A2a	Faeces	yes	yes
IBE-BC1417	41.21845	-3.29172	Tajo	2015	B	Faeces	yes	yes
IBE-BC1432	41.10345	-3.48929	Tajo	2015	A2b	Faeces	yes	yes
IBE-BC1436	41.16917	-3.37749	Tajo	2015	B	Faeces	yes	yes
IBE-BC1442	40.32822	-7.58676	Tajo	2015	A1	Faeces	yes	yes
IBE-BC1480	40.34883	-7.56329	Tajo	2015	A2a	Faeces	yes	yes
IBE-BC1507	40.44262	-7.51313	Mondego	2015	A1	Faeces	yes	yes
IBE-BC1543	40.35643	-7.55865	Tajo	2015	A1	Faeces	yes	yes

Table S2. Mitochondrial genetic diversity statistics of the cytochrome *b* (Cytb) and D-loop fragments of *Neomys anomalus* calculated for all the samples and for the four main mitochondrial phylogroups (A and B). N = number of sequences; S = number of segregating sites; h = number of haplotypes; Hd = haplotype diversity; π = nucleotide diversity per site; Ø = Watterson's estimator of nucleotide diversity per site.

	Cytb (All)	Cytb (A)	Cytb (B)	D-loop (All)	D-loop (A)	D-loop (B)
N	132	78	54	103	65	38
S	64	49	13	82	65	45
h	50	35	15	65	41	24
Hd	0.96	0.96	0.83	0.98	0.98	0.96
π	0.0083	0.0068	0.0020	0.0455	0.0386	0.0362
Ø	0.0138	0.0116	0.0033	0.0511	0.0439	0.0338

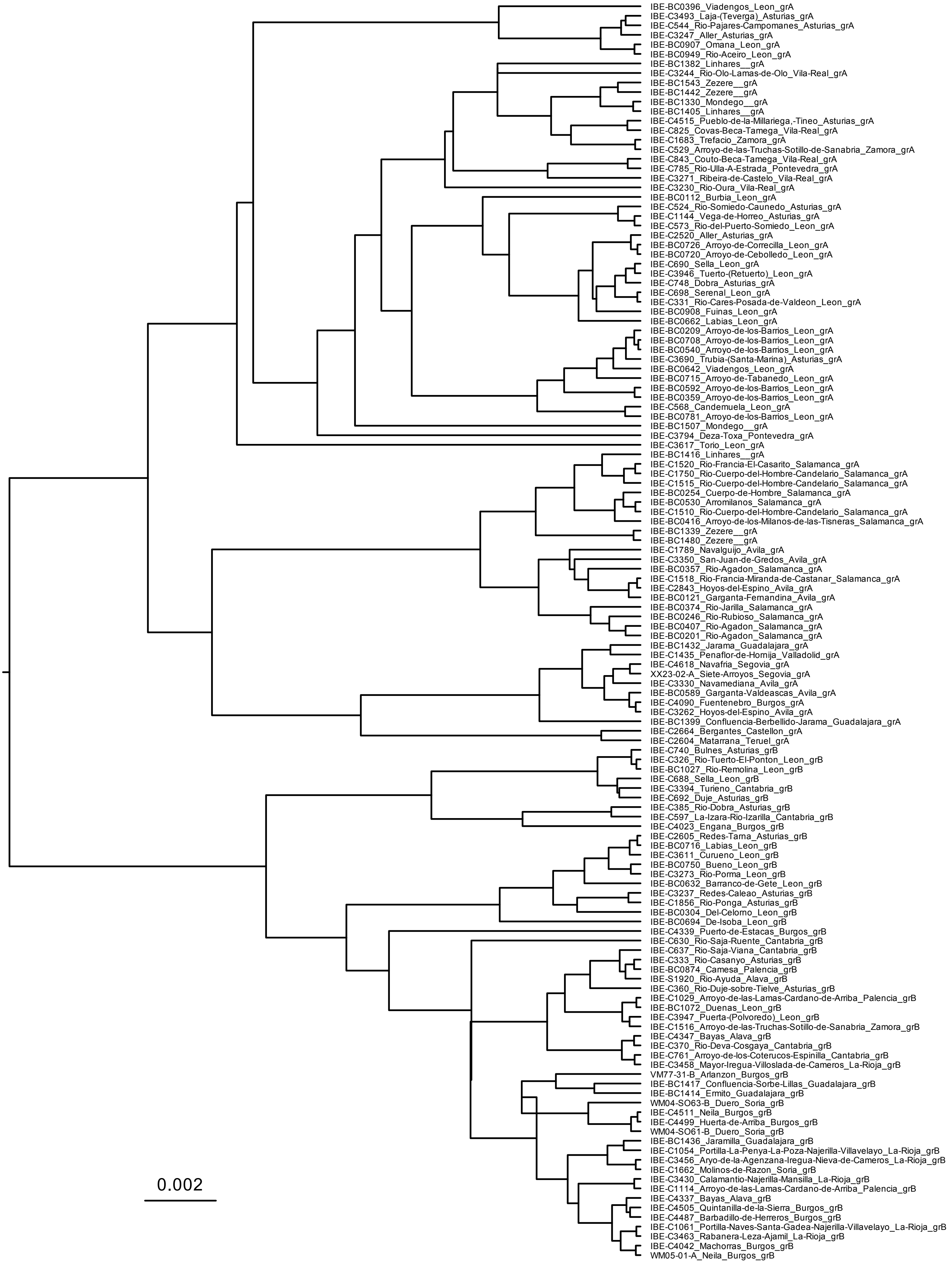

Figure S1. Beast tree with tips labeled with sample codes and locality information (locality and province). The scale bar is in substitutions per position.

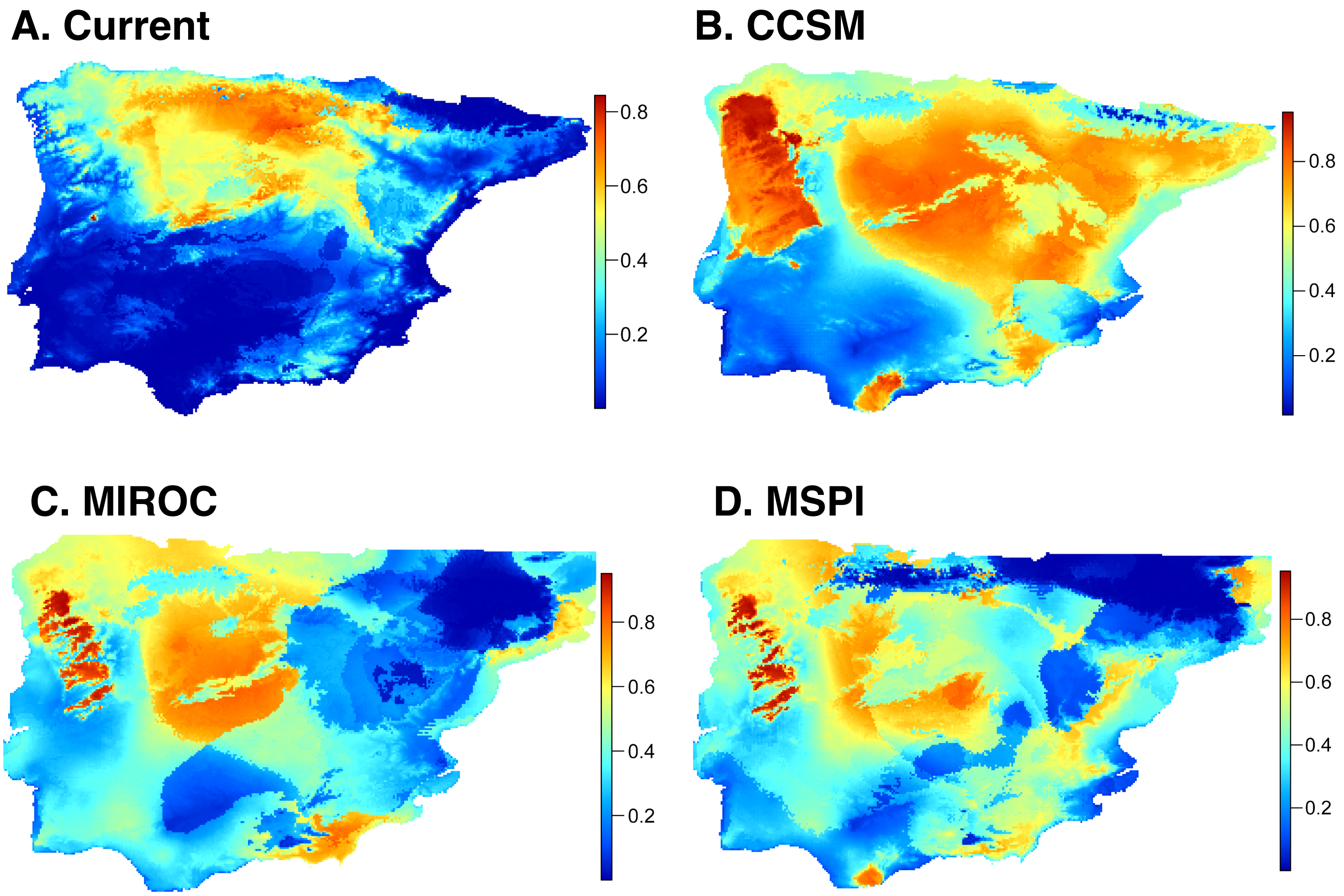

Figure S2. Projection of the species distribution model to the current climate scenario (A) and to the Last Glacial climatic models: CCSM (B), MIROC (C) and MSPI (D).
